# Variation in leaf traits at different altitudes reflects the adaptive strategy of plants to environmental changes

**DOI:** 10.1002/ece3.6519

**Published:** 2020-07-15

**Authors:** Wensheng Liu, Li Zheng, Danhui Qi

**Affiliations:** ^1^ College of Life Science and Technology Central South University of Forestry and Technology Changsha China; ^2^ Southwest Forestry University Kunming China

**Keywords:** altitudinal gradient, anatomical structure, plant life form, stomatal density, the Yulong Mountains

## Abstract

Leaf anatomical traits play key roles in plant functions and display evolutionary adaptive changes to suit the surrounding environment. To reveal the adaptive mode and mechanisms of plants in response to global warming, we analyzed leaf morphology and anatomical structures in three different species, *Epilobium amurense* Hausskn., *Pedicularis densispica* Franch., and *Potentilla fulgens* Wall. ex Hook., growing along an elevational gradient (3,000–4,600 m) in the Yulong Mountains. The results showed leaf length and width decreased, whereas leaf thickness increased with increasing altitude in all three species. Thickness of leaf upper epidermis, lower epidermis, palisade and spongy mesophyll, and main vein increased with rising altitude. Stomatal density in each species increased with rising elevation. These results illustrate that plants can adapt to the environmental changes that accompany high altitudes by decreasing leaf area and increasing leaf thickness, mesophyll tissue thickness, and stomatal density. Such morphological and anatomical plasticity would lead to lower transpiration rates, enhanced internal temperature and water status, and improved photosynthetic capability.

## Graphical abstract

With increasing altitude, leaf length and width decreased in all three species, whereas leaf thickness increased. Stomatal density in each species also increased with increasing altitude; however, both stoma length and width decreased. Thickness of the leaf upper epidermis, lower epidermis, palisade and spongy mesophyll, and midrib increased with increasing altitude.

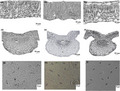

## 1 INTRODUCTION

Leaves are directly involved in photosynthetic carbon fixation, respiration, and transpiration, and are the most sensitive parts to climatic changes (Carlson, Adams, & Holsinger, 2016; Chen, Niklas, Chen, & Guo, 2012). Leaf traits, which vary across different climatic conditions, play key roles in plant functions under its environment and can provide insight into the adaptive evolutionary changes made to suit the surrounding conditions (Guo, Ma, Yuan, & Wang, 2017; Körner, Bannister, & Mark, 1986; Li & Bao, 2014; Tian, Yu, He, & Hou, 2016; Wang et al., 2014, 2015). The analysis of how leaf traits, such as morphology and anatomical structures, change along climatic gradients assists in predicting how plants will cope with future global climatic changes (Guo et al., 2017; Milla, Giménez‐Benavides, Escudero, & Reich, 2009). A number of studies have investigated variations in leaf morphology and anatomical structures along climatic gradients of longitude, latitude, and altitude; however, these studies primarily focused on variations in general leaf anatomy between many species (Guo et al., 2017; Tian et al., 2016). Leaf adaptations among populations of a single species growing in different climatic conditions remain poorly comprehended, and a better understanding would help to clarify the mechanism of plants to environmental changes (Olsen, Caudle, Johnson, Baer, & Maricle, 2013).

Elevation gradients provide natural climatic variation in which key environmental factors that affect plant growth and development, including atmospheric temperature, CO_2_ partial pressure, solar irradiance, and rainfall, change considerably within a confined area. Along these gradients, plants would adjust their performances to adapt to the associated climatic conditions, which would reflect the projected trends of global climatic changes, to some extent (Read, Moorhead, Swenson, Bailey, & Sanders, 2014). Thus, elevation gradients are ideal platforms to study plant responses to global climatic changes (Li & Bao, 2014; Read et al., 2014; Wang et al., 2014).

Plants can exhibit phenotypic and anatomical plasticity to optimize their resource utilization strategy under various environmental conditions (Olsen et al., 2013; Read et al., 2014; Wang et al., 2011, 2014). Plasticity in morphology and anatomical structures is an effective means for plants to respond appropriately to long‐term climate change (Guo et al., 2017). As elevation ascended, the air temperature decreased abruptly, which is the major environmental factor affecting plant survival, distribution, growth, and reproduction. Common plant adaptive strategies to tolerate low temperature involve minimizing heat loss and the exposure of internal tissues to low temperatures. Thus, plants tend to decrease leaf area and increase thickness of leaf, upper and lower epidermises, and palisade and spongy mesophyll tissues to increase boundary layer resistance with ascending altitudes (Körner, 2003; Körner et al., 1986; Wang, Yu, et al., 2016). However, some studies have presented contrasting results, such as increasing leaf area in *Picea schrenkiana* var. *tianschanica* Rupr. (Zhang, Ma, Sun, & Chen, 2010), *Kobresia capillifolia* (Decne.) C. B. Clarke (Zhong et al., 2014), and *Campylotropis polyantha* (Franch.) Schindl. (Li & Bao, 2014), decreasing leaf thickness and mesophyll tissue thickness in *C. polyantha* (Li & Bao, 2014), *Picea likiangensis* (Franch.) Pritz. (Tang, Jiang, Feng, Cheng, & Huang, 2015), *Saussurea superba* Anthony (Sun et al., 2016), and *Meconopsis integrifolia* (Maxim.) French. (Liu et al., 2018) with rising elevation. These studies illustrate that plants have complicated adaptive means. Further studies are required to reveal the ways to respond to increasing altitudes and adaptive mechanisms for plants.

Stomata are small pores on the surfaces of plant leaves that act as turgor‐operated valves to control the exchange of gases (e.g., water vapor and CO_2_) between plant tissues and the atmosphere. Thus, stomata facilitate plant respiration and transpiration and play major roles in the regulation of water and carbon cycling (Wang et al., 2014; Wen, Chen, Teng, Zhang, & Wang, 2018). The size and distribution of stomata are key adaptive traits which vary greatly in response to environmental changes. As altitude increases, air temperature, and CO_2_ and O_2_ partial pressure decrease, stomatal density has been found to change along these gradients, but patterns are inconsistent (Sun et al., 2016; Wang et al., 2014; Yang et al., 2014). For example, increased stomatal density with increasing altitude was observed for six pteridophyte species from the Bolivian Andes to increase the absorption of oxygen and CO_2_; however, no correlation was apparent for five other species (Kessler, Siorak, Wunderlich, & Wegner, 2007). In contrast, decreased stomatal density in a mixture of 12 species of trees, shrubs, and herbaceous plants was found with increasing altitude in the Southern Alps, New Zealand (Körner et al., 1986). Hence, further study is necessary to understand the adaptive mechanism of stomatal density in relation to climatic change.

The Yulong Mountains are located in Northwest Yunnan, China, which is a hotspot characterized by high biodiversity (Feng, Wang, Xu, Yang, & Fang, 2006; Huang et al., 2017). The mountains are situated at low latitudes with a subtropical climate and great disparity in altitude, which creates an ideal study site for vertical biodiversity and ecological adaptation of plants (Tang et al., 2015; Wang, Yu, et al., 2016). *Epilobium amurense* Hausskn., *Pedicularis densispica* Franch., and *Potentilla fulgens* Wall. ex Hook. are three herb species that occur across a large elevation range in the Yulong Mountains. *E. amurense* and *P. fulgens* are perennials, and *P. densispica* is an annual plant. The three plants are three dominant species in their communities and play important roles in water and soil conservation of the mountains. In this study, the leaf morphology and anatomical structures of these three species were studied to assess how leaf anatomical structures and stomatal traits change with increasing altitude. The aim is to reveal the mechanisms of these species to cope with varying environmental conditions.

## 2 MATERIALS AND METHODS

### 2.1 Study sites

The Yulong Mountains are located at 27° 10′–27° 40′N and 100° 10′–100° 20′E; the longest north–south length is 35 km, and the longest west–east width is 12 km. The highest altitude of these mountains is 5,596 m, and the distance between the highest and the lowest point is 3,846 m. The mean annual precipitation is 935 mm, and the mean annual temperature is 12.79°C. The vegetation types are comprised of *Pinus yunnanensis* Franch. forests, *P. likiangensis* forests, *Larix potaninii* Batalin forests, and *Abies georgei* Orr forests from 2,400 m to 3,800 m; and alpine scrub (including *Quercus aquifolioides* Rehd. et Wils.) and alpine meadow (including *Kobresia tunicate* Hand.) above 3,800 m (Feng et al., 2006; Huang et al., 2017).

### 2.2 Field sampling

Nine sample plots were defined at elevation intervals of 200 m located on the southern slopes of the Yulong Mountains along an elevation gradient ranging within 3,000–4,600 m. In these plots, Plot 1, Plot 2, Plot 3, and Plot 4 range from 3,000 to 3,600 m in altitude and are covered by *Pinus yunnanensis* forests. Plot 5 and Plot 6 are located at 3,800 m and 4,000 m and are distributed within *Quercus aquifolioides* scrub. Plot 7, Plot 8, and Plot 9 are situated at 4,200–4,600 m and are covered by alpine meadows. *E. amurense*, *P. densispica*, and *P. fulgens* are three plant species distributed in these plots, and the plant height of these species ranges within 62.31–12.76 cm, 23.21–9.16 cm, and 33.92–8.93 cm (Table 1).

**TABLE 1:** Plot information of *Epilobium amurense*, *Pedicularis densispica*, and *Potentilla fulgens*

Plot name	Elevation (m)	Mean slope	Aspect	Plant height (cm)			Vegetation type
				*E. amurense*	*P. densispica*	*P. fulgens*	
P1	3,000	35°	SE25°	62.31 ± 14.67	–	33.92 ± 7.48	*Pinus yunnanensis* forests
P2	3,200	30°	SE27°	59.91 ± 11.39	23.21 ± 2.52	28.5 ± 11.97	*P. yunnanensis* forests
P3	3,400	28°	SE31°	56.77 ± 10.14	21.26 ± 4.16	23.79 ± 9.81	*P. yunnanensis* forests
P4	3,600	26°	SE30°	40.86 ± 9.37	15.79 ± 4.84	13.52 ± 2.51	*P. yunnanensis* forests
P5	3,800	22°	SE28°	34.45 ± 8.3	12.25 ± 2.52	10.49 ± 2.26	*Quercus aquifolioides* scrub
P6	4,000	24°	SE15°	33.06 ± 7.8	10.66 ± 3.22	8.93 ± 3.53	*Q. aquifolioides* scrub
P7	4,200	15°	SE19°	19.63 ± 5.4	10.16 ± 2.07	–	Alpine meadow
P8	4,400	15°	S	14.87 ± 3.12	9.16 ± 1.74	–	Alpine meadow
P9	4,600	10°	S	12.76 ± 2.35	5.89 ± 1.45	–	Alpine meadow

Samples were collected in 2015 between July and August when plants were flowering and fruiting. At each sampling plot, a sampling quadrat (50 m × 50 m) was delimited. In each sampling quadrat (population), five fully expanded sun leaves were collected from five individuals each of *E. amurense*, *P. densispica*, and *P. fulgens* for analysis of leaf morphology and anatomical structures, respectively, except where there were no corresponding species in the plot. In total, leaves from 23 populations were sampled, including nine *E. amurense* populations (3,000–4,600 m), eight *P. densispica* populations (3,200–4,600 m), and six *P. fulgens* populations (3,000–4,000 m, Table 1).

In order to measure leaf anatomical traits, rectangular pieces (1 cm × 0.5 cm) that included the midrib (main vein) and a portion of the lamina were cut out from the leaves and fixed in formalin–acetic acid–alcohol solution (FAA, 5 ml of 37% formalin, 5 ml of glacial acetic acid, and 90 ml of 50% ethanol mixed with 5 ml glycerin). Twenty‐five pretreated leaves from each species from each population were randomly selected to measure leaf anatomical traits.

### 2.3 Measurement of leaf size and anatomical traits

Leaf samples were progressively dehydrated in a series of ethanol solutions (50%–100%) and infiltrated with warm paraffin (56–58°C). Leaf sections 8–10 μm in size were obtained using a rotary microtome (Leica, RM2255, Germany). Leaf sections on slides were stained with safranin and fast green (1% aqueous safranin and 0.5% fast green in 95% ethanol).

Leaf length and leaf width were determined by vernier caliper (accuracy 0.02 mm). Leaf thickness (μm), thickness of the upper and lower epidermis (μm), palisade mesophyll thickness (μm), spongy mesophyll thickness (μm), and main vein thickness (μm) were analyzed with a light microscope (Motic B5 Professional Series) at a magnification of 400× to measure by stained sections. A total of 25 data points for leaf thickness, palisade mesophyll thickness, and spongy mesophyll thickness were obtained for each sampling population of each species.

Stomatal traits were measured in leaf sections avoiding leaf veins. Epidermal images were taken from the leaves by the sodium hypochlorite solution method (Pan, Lu, & Wen, 1990). Stomatal length and density were obtained using Motic Microscopic Image System (Motic B5 Professional Series). In each image, we determined stoma length and width (μm) by measuring five randomly selected stomata. For the measurement of stomatal density (stomata/mm^−2^), the number of stomata per unit area (mm^−2^) was counted within the images at a magnification of 400×. A total of 25 data points for stoma length/width and 25 data points for stomatal density were obtained for each sampling population of each species.

### 2.4 Plasticity index

Plasticity index (PI) was calculated according to the formula of Gratani, Covone, and Larcher (2006).

PI=1‐x/X

where *x* denotes the lowest average value of all the populations and *X* denotes the highest average value of all the populations of the corresponding species.

### 2.5 Data analysis

Regression analysis between leaf morphological and anatomical traits and altitudes, and differences in leaf traits among plants at different altitudes were analyzed using SPSS 13.0 (IBM Corp., Chicago, IL, USA). Prior to statistical analysis, the Kolmogorov–Smirnov test was applied to assess data normality and the Levene test was applied for homogeneity of variances. Data were log‐transformed as needed to ensure that the original datasets followed a normal distribution and equal variances in this study. The differences among the plant populations were analyzed using a one‐way analysis of variance (ANOVA). If variances were equal, least significant difference (LSD) tests were used to make multiple comparisons. If variances were unequal, Tamhane's T2 tests were used. *p* < .05 denotes significant difference. Final data presented are the means ± standard deviation (*SD*). Graphs were drawn using SigmaPlot 12.5 (Systat Software Inc., San Jose, CA, USA).

## 3 RESULTS

### 3.1 Leaf morphology

**FIGURE 1:**
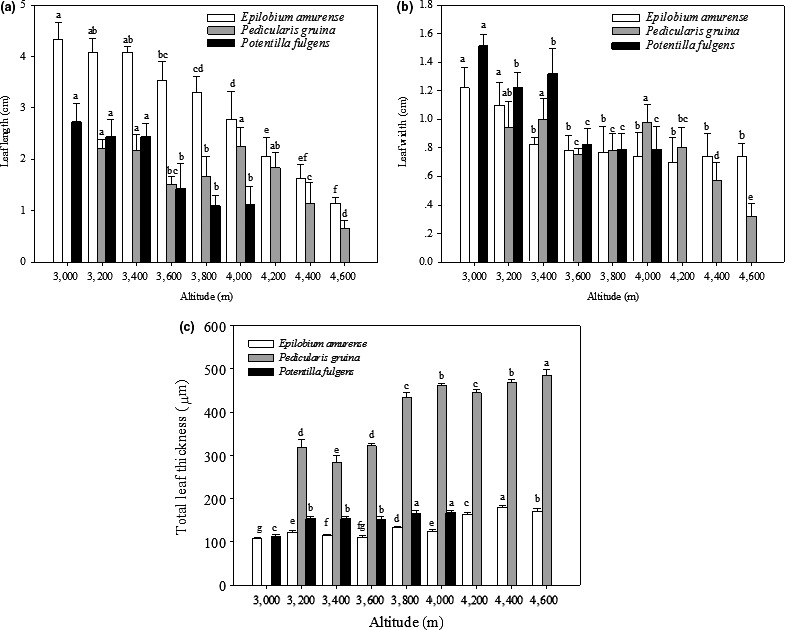
Leaf length (a), width (b), and thickness (c) of *E. amurense*, *P. densispica*, and *P. fulgens* growing along an altitudinal gradient in the Yulong Mountains. Data are presented as means ± *SE*. Different letters indicate significant differences among elevations based on an LSD test (*n* = 5, *p* < .05)

The results showed that the leaf length and width in *E. amurense* (leaf length: *R* ^2^ = 0.962, *p* = .000; leaf width: *R* ^2^ = 0.664, *p* = .007, respectively), *P. densispica* (leaf length: *R* ^2^ = 0.566, *p* = .031; leaf width: *R* ^2^ = 0.607, *p* = .023, respectively), and *P. fulgens* (leaf length: *R* ^2^ = 0.891, *p* = .005; leaf width: *R* ^2^ = 0.834, *p* = .011, respectively) were negatively correlated with altitude (Figure 1a, b). Measurement of leaf thickness revealed the reverse trend, whereby the leaf thickness in each species (*E. amurense*, *R* ^2^ = 0.771, *p* = .002; *P. densispica*, *R* ^2^ = 0.815, *p* = .002; *P. fulgens*, *R* ^2^ = 0.687, *p* = .042) was positively correlated with altitude (Figure 1c).

### 3.2 Epidermal layer

**FIGURE 2:**
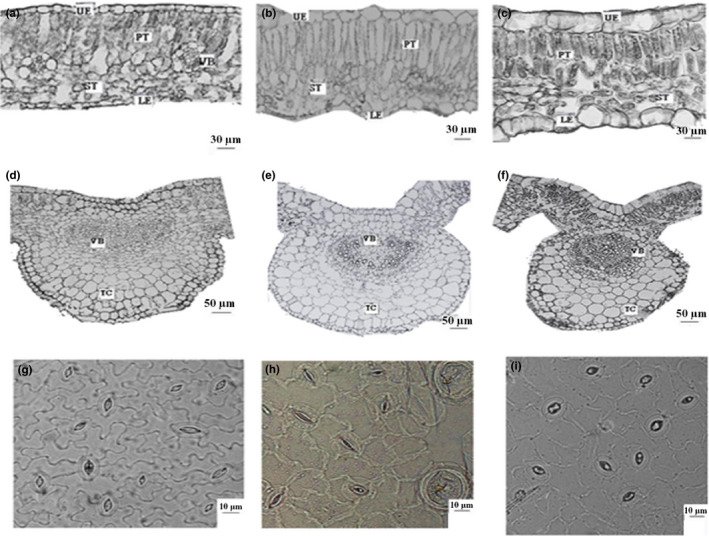
Cross sections of leaf blade of *E. amurense* (a, 4,200 m), *P. densispica* (b, 4,600 m), and *P. fulgens* (c, 3,600 m); cross sections of main veins of *E. amurense* (d, 4,200 m), *P. densispica* (e, 4,600 m), and *P. fulgens* (f, 3,600 m); and stomatic characteristic of lower epidermis of *E. amurense* (g, 4,200 m), *P. densispica* (h, 4,600 m), and *P. fulgens* (i, 3,600 m). UE, upper epicuticle; LE, lower epicuticle; PT, palisade tissue; ST, spongy tissue; VB, vascular bundle

**FIGURE 3:**
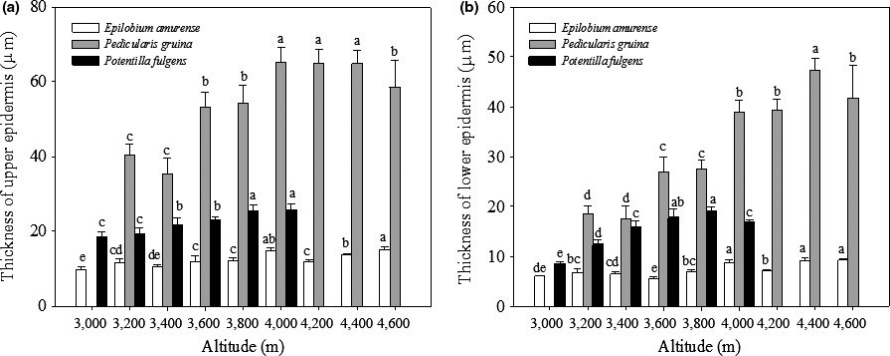
Thickness of the leaf upper epidermis (a) and lower epidermis (b) of *E. amurense*, *P. densispica*, and *P. fulgens* growing along an altitudinal gradient in the Yulong Mountains. Data are presented as means ± SE. Different letters indicate significant differences among elevations based on an LSD test (*n* = 5, *p* < .05)

Epidermal layers were shown in Figure 2. Regression analysis showed that the thicknesses of upper and lower epidermises in *E. amurense* (upper epidermis: *R* ^2^ = 0.696, *p* = .005; lower epidermis: *R* ^2^ = 0.651, *p* = .009), *P. densispica* (upper epidermis: *R* ^2^ = 0.671, *p* = .013; lower epidermis: *R* ^2^ = 0.889, *p* = .000), and *P. fulgens* (upper epidermis: *R* ^2^ = 0.966, *p* = .000; lower epidermis: *R* ^2^ = 0.748, *p* = .026) (Figure 3a, b) were positively correlated with altitude.

### 3.3 Stomatal traits

**FIGURE 4:**
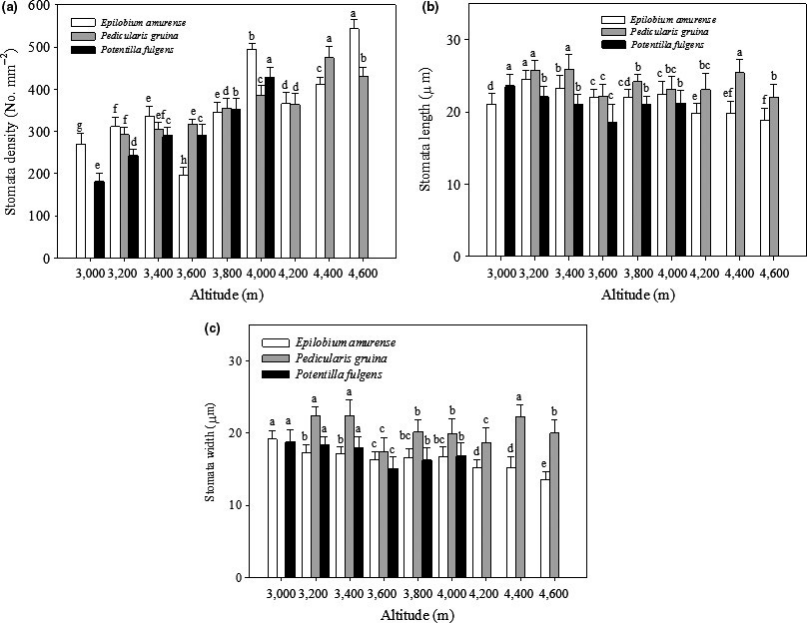
Stomatal density (a), length (b), and width (c) of *E. amurense*, *P. densispica,* and *P. fulgens* growing along an altitudinal gradient in the Yulong Mountains. Data are presented as means ± *SE*. Different letters indicate significant differences among elevations based on an LSD test (*n* = 25, *p* < .05)

Regression analysis showed that stomatal density in *E. amurense* (*R* ^2^ = 0.552, *p* = .022), *P. densispica* (*R* ^2^ = 0.835, *p* = .002), and *P. fulgens* (*R* ^2^ = 0.953, *p* = .001) was positively correlated with altitude (Figure 4a).

Regression analysis also showed that stoma length in *E. amurense* (*R* ^2^ = 0.557, *p* = .021) was negatively correlated with altitude, whereas no correlation was observed in *P. densispica* (*R* ^2^ = 0.247, *p* = .210) and *P. fulgens* (*R* ^2^ = 0.324, *p* = .239) (Figure 4b). Stoma width in each species was negatively correlated with altitude (*E. amurense*: *R* ^2^ = 0.856, *p* = .000; *P. densispica*: *R* ^2^ = 0.052, *p* = .588; *P. fulgens, R* ^2^ = 0.514, *p* = .109) (Figure 4c).

### 3.4 Mesophyll

**FIGURE 5:**
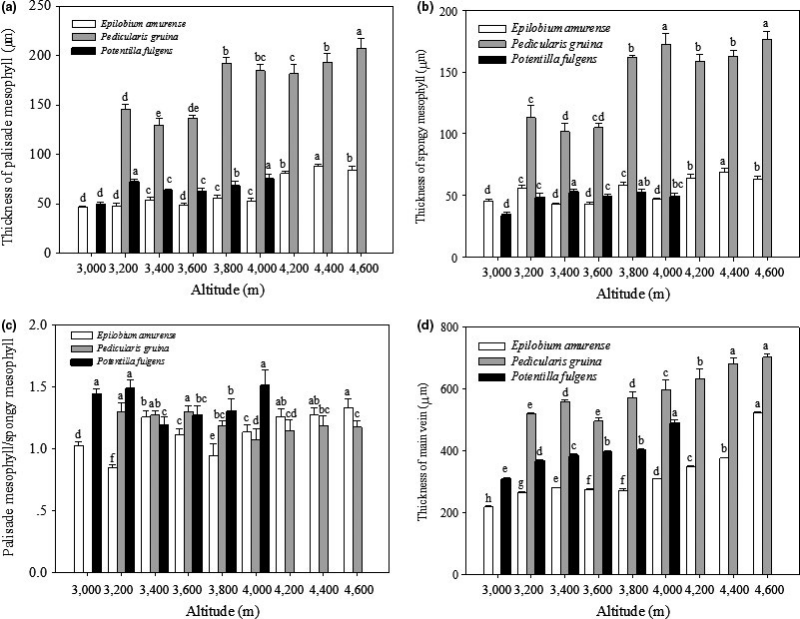
Thickness of the leaf palisade mesophyll (a), spongy mesophyll (b), ratio of palisade mesophyll thickness to spongy mesophyll thickness (c), and main vein (d) of *E. amurense*, *P. densispica*, and *P. fulgens* growing along an altitudinal gradient in the Yulong Mountains. Data are presented as means ± *SE*. Different letters indicate significant differences among elevations based on an LSD test (*n* = 5, *p* < .05)

Regression analysis showed that the thickness of palisade mesophyll tissue in *E. amurense* (*R* ^2^ = 0.773, *p* = .002) and *P. densispica* (*R* ^2^ = 0.752, *p* = .005) was positively correlated with altitude, whereas that in *P. fulgens* (*R* ^2^ = 0.440, *p* = .151) did not display a correlation (Figure 5a).

The thickness of spongy mesophyll tissue in *E. amurense* (*R* ^2^ = 0.500, *p* = .033) and *P. densispica* (*R* ^2^ = 0.724, *p* = .007) showed a positive correlation with altitude, whereas that in *P. fulgens* (*R* ^2^ = 0.416, *p* = .166) did not display a correlation (Figure 5b).

The ratio of palisade mesophyll thickness to spongy mesophyll thickness showed a positive correlation with altitude in *E. amurense* (*R* ^2^ = 0.489, *p* = .036), but showed no correlation in *P. densispica* (*R* ^2^ = 0.437, *p* = .074) and *P. fulgens* (*R* ^2^ = 0.001, *p* = .957) (Figure 5c).

Regression analysis showed that the thickness of the leaf main vein in *E. amurense* (*R* ^2^ = 0.766, *p* = .002), *P. densispica* (*R* ^2^ = 0.856, *p* = .001), and *P. fulgens* (*R* ^2^ = 0.856, *p* = .008) was positively correlated with altitude (Figure 5d).

Calculation of anatomical plasticity indices showed that the traits of leaf length, leaf width, lower epidermis thickness, and stomatal density each had a high plasticity index (Table 2). By contrast, the traits of leaf thickness, upper epidermis thickness, palisade mesophyll thickness, spongy mesophyll thickness, palisade and spongy mesophyll combined thickness, main vein thickness, stoma length, and stoma width each had a low plasticity index. In addition, the annual plant species (*P. densispica*) displayed a lower anatomical plasticity for main vein thickness, stomatal density, stoma length, and stoma width than the perennial herb species (*E. amurense* and *P. fulgens*) (Table 2).

**TABLE 2:** The anatomical plasticity index of leaves of *Epilobium amurense*, *Pedicularis densispica*, and *Potentilla fulgens*

Trait	*E. amurense*	*P. densispica*	*P. fulgens*
Leaf length (LL)	0.74	0.71	0.60
Leaf width (LW)	0.43	0.68	0.48
Leaf thickness (LT)	0.40	0.41	0.33
Thickness of upper epidermis (TUE)	0.35	0.46	0.28
Thickness of lower epidermis (TLE)	0.40	0.63	0.55
Thickness of palisade (TP)	0.47	0.38	0.34
Thickness of spongy (TS)	0.38	0.42	0.35
Thickness of palisade and spongy (PS)	0.36	0.18	0.21
Thickness of main vein (TM)	0.58	0.29	0.37
Stomatal density (SD)	0.64	0.38	0.58
Stoma length (SL)	0.23	0.15	0.21
Stoma width (SW)	0.29	0.22	0.20

## 4 DISCUSSION

### 4.1 Leaf size

Leaf size comprehensively reflects the functions of leaf, and it is an important plant trait for plants to adapt to environment. In the present study, leaf size (denoted as leaf length and width) in each of the three plant species, *E. amurense*, *P. densispica*, and *P. fulgens*, decreased with altitude, indicating that plants growing at higher altitudes have smaller leaves than those at lower altitudes. The same leaf size response has also been observed in other studies (Körner, 2003; Körner et al., 1986; Liu et al., 2018; Wang, Qi, Liu, & Liang, 2016; Zhang, Wang, Li, Xu, & Liu, 2014). The main reason explaining this pattern is that plant growth is limited by the low minimum or mean annual temperatures, high levels of solar irradiation, and wind exposure typical of high‐altitude environments, and the leaf size decreases with rising elevation accordingly. In addition, small leaf size can also reduce the absorption of solar energy and the rate of evapotranspiration to decrease the damage from high‐level ultraviolet irradiation and strong wind exposure (Tian et al., 2016). In summary, small leaf size is a significant adaptation to cope with low temperature, high levels of solar irradiation, and wind exposure at high altitudes.

### 4.2 Anatomical structures

The leaf epidermis is an important protective tissue that protects leaves from injury from high rates of water transpiration and high light irradiation at high altitudes. The results of this study showed that leaf thickness and thickness of the upper and lower epidermis increased with enhancing altitudes in the three studied species, which is consistent with other studies (Körner, 2003; Körner et al., 1986; Sun et al., 2016; Zhang et al., 2014). It illustrated that increasing epidermal thickness and leaf thickness are important adaptive traits to environmental conditions at high altitudes. Thicker leaves and epidermis can provide a greater buffer between inner leaf temperature and outer environmental temperature and keep higher internal temperature, which would contribute to maintaining normal physiological activity for plants under low temperatures at higher altitude. In addition, thicker leaves and leaf epidermises can reduce the damage inflicted by the high‐level ultraviolet irradiation present at high altitudes (Ma et al., 2012). It can also facilitate greater water storage and is evolutionarily favorable for efficient water use and reducing transpiration (Guo et al., 2017).

Palisade and spongy mesophyll are important tissues for photosynthesis as they harbor the chloroplasts where the photosynthetic reactions mainly occur. Plant species with a thicker mesophyll may have better photosynthetic capacity (Liu et al., 2018). The present study showed that the thicknesses of palisade and spongy mesophyll in the three species increased with rising altitudes. This trend is the same as other studies (Körner, 2003; Körner et al., 1986; Shi, Wang, & Wang, 2009; Sun et al., 2016; Wang, Qi, et al., 2016). Thicker palisade mesophyll contributes to maintaining higher internal temperature and water status in the leaf, which may be an adaptation to the low temperatures and low water availability at high altitudes (Körner, 2003). In addition, thicker palisade and spongy mesophyll play a significant role in reducing tissue damage by abundant solar irradiation and maintaining high levels of photosynthesis. Overall, it suggests that thicker leaf, epidermis, and mesophyll tissue contribute to adapt to low temperatures, low water availability, and high‐intensity ultraviolet irradiation present at high altitudes for plants.

The main vein of the leaf is an important tissue because it provides water to the leaf blade. The present study showed that main vein thickness of the three species increased with ascending altitudes. This pattern was also reported in other studies (Wang, Qi, et al., 2016). It revealed that this is an important adaptive trait, because a large main vein creates a strong water transport capacity for plants, which enables the plants to maximize hydraulic conductance and to maximize investment in xylem structures at high elevation accompanied by low temperatures. Thus, an increasing main vein thickness is an adaptive strategy of plants to enhance water transport under low temperatures at high altitudes.

### 4.3 Stomatal variation

Stomata are essential connections between the internal leaf space and the external atmosphere, and stomatal density is an important trait that directly regulates the exchange of CO_2_ and H_2_O (Wang et al., 2014; Yang et al., 2014). The present study showed that stomatal density of the three studied species increased with enhancing altitude, which is the same as in other studies (Körner et al., 1986; Sun et al., 2016; Wang et al., 2014; Yang et al., 2014). The relationship between stomatal density and altitude could be explained by the CO_2_ availability theory (Pato & Obeso, 2012). The theory assumed that the partial pressure of CO_2_ (P_CO2_) had a significant effect on stomatal density. Since CO_2_ and O_2_ become thinner with rising altitude, plants may increase stomatal density to enhance their gas absorption capabilities. In high densities, stomata facilitate a rapid increase in stomatal conductance that maximizes CO_2_ diffusion for photosynthesis under favorable environmental conditions, which would contribute to higher plant survival at high altitudes. In addition to CO_2_ availability, stomatal density was also affected by light intensity, which influences epidermal cell expansion and was shown to be positively correlated with stomatal density (Hovenden & Vander, 2006). Thus, a higher stomatal density is a functional response of plants to low CO_2_ and high‐intensity light at high elevations.

In the present study, although the annual and perennial herb species exhibited the same adaptive patterns in plant traits along the elevational gradients, the annual plant species (*P. densispica*) displayed lower anatomical plasticity for main vein thickness, stomatal density, stoma length, and stoma width than the perennial herb species (*E. amurense* and *P. fulgens*) (Table 2). Guo et al. (2017) assessed leaf thickness of plants of different life forms (trees, shrubs, perennial forbs, perennial grasses, and annual grasses) along a large‐scale aridity gradient and found annual grasses had the least plasticity along the precipitation gradient. These studies illustrated annual plants had lower anatomical plasticity. We ascribed it to the annual plants growing mainly during rainy and warm seasons in the present study and use seasonal precipitation and heat efficiently in the extreme regions. Further studies are needed to compare the differences in water use efficiency along temperature gradients in annual versus perennial species.

## CONFLICT OF INTEREST

None declared.

## AUTHOR CONTRIBUTION

**Wensheng Liu:** Resources (equal); Writing‐review & editing (equal). **Li Zheng:** Writing‐original draft (equal). **Danhui Qi:** Data curation (equal); Formal analysis (equal); Investigation (equal).

## Supporting information

Supinfo

## Data Availability

All data supporting these findings have been made available within the manuscript.
